# Automatic multiple zebrafish larvae tracking in unconstrained microscopic video conditions

**DOI:** 10.1038/s41598-017-17894-x

**Published:** 2017-12-14

**Authors:** Xiaoying Wang, Eva Cheng, Ian S. Burnett, Yushi Huang, Donald Wlodkowic

**Affiliations:** 1 School of Engineering, RMIT University, Melbourne, 3000 Australia; 2 Faculty of Engineering & Information Technology, University of Technology Sydney, 2007 Sydney, Australia; 3 School of Science, RMIT University, Melbourne, 3083 Australia

## Abstract

The accurate tracking of zebrafish larvae movement is fundamental to research in many biomedical, pharmaceutical, and behavioral science applications. However, the locomotive characteristics of zebrafish larvae are significantly different from adult zebrafish, where existing adult zebrafish tracking systems cannot reliably track zebrafish larvae. Further, the far smaller size differentiation between larvae and the container render the detection of water impurities inevitable, which further affects the tracking of zebrafish larvae or require very strict video imaging conditions that typically result in unreliable tracking results for realistic experimental conditions. This paper investigates the adaptation of advanced computer vision segmentation techniques and multiple object tracking algorithms to develop an accurate, efficient and reliable multiple zebrafish larvae tracking system. The proposed system has been tested on a set of single and multiple adult and larvae zebrafish videos in a wide variety of (complex) video conditions, including shadowing, labels, water bubbles and background artifacts. Compared with existing state-of-the-art and commercial multiple organism tracking systems, the proposed system improves the tracking accuracy by up to 31.57% in unconstrained video imaging conditions. To facilitate the evaluation on zebrafish segmentation and tracking research, a dataset with annotated ground truth is also presented. The software is also publicly accessible.

## Introduction

As zebrafish (Danio rerio) larvae have emerged as a vertebrate and mammal model for many biomedical applications including screening for biochemical abnormalities^1^ and behavioral science investigations^2^, the tracking of the larvae has emerged as a challenge. Typical manual tracking approaches are tedious, commonly requiring significant periods of manual observation and labelling of the image features to represent the activity for a single experimental task^3^. Furthermore, as a subjective manual task, the results are difficult to reliably repeat and reproduce. Recent research attention has therefore focused on the development of automatic multiple zebrafish larvae tracking systems due to the increased availability of digital microscopy and video storage systems.

Many automatic single and multiple tracking systems have been recently developed for adult zebrafish^1,4–9^, such as the state-of-the-art based on deep learning^7^, particle filtering^9^, and the well-known idTracker^5^, reporting outstanding tracking performance for adult zebrafish. However, the locomotive characteristics of zebrafish larvae are dramatically different from adult zebrafish. Adult fish are continually swimming, whilst zebrafish larvae can display little or no movement over time^8,10^, thus their dynamic responses can be imbalanced. Zebrafish larvae can exhibit a mean proportion of activities less than 0.075 over time, according to the statistics reported in^8^. This is the first primary cause of tracking failure in these systems and traditional statistical tests based on movement features to track and analyse larvae behaviour. Moreover, the intensity contrast between adult fish with the water background is also greater than that for zebrafish larvae, due to the transparent larvae body peripheral. However, both the adult zebrafish tracking systems in^5,7^ are based on the assumption of high intensity contrast, which is another common and required imaging condition constraint for existing zebrafish tracking systems.

**Figure 1:**
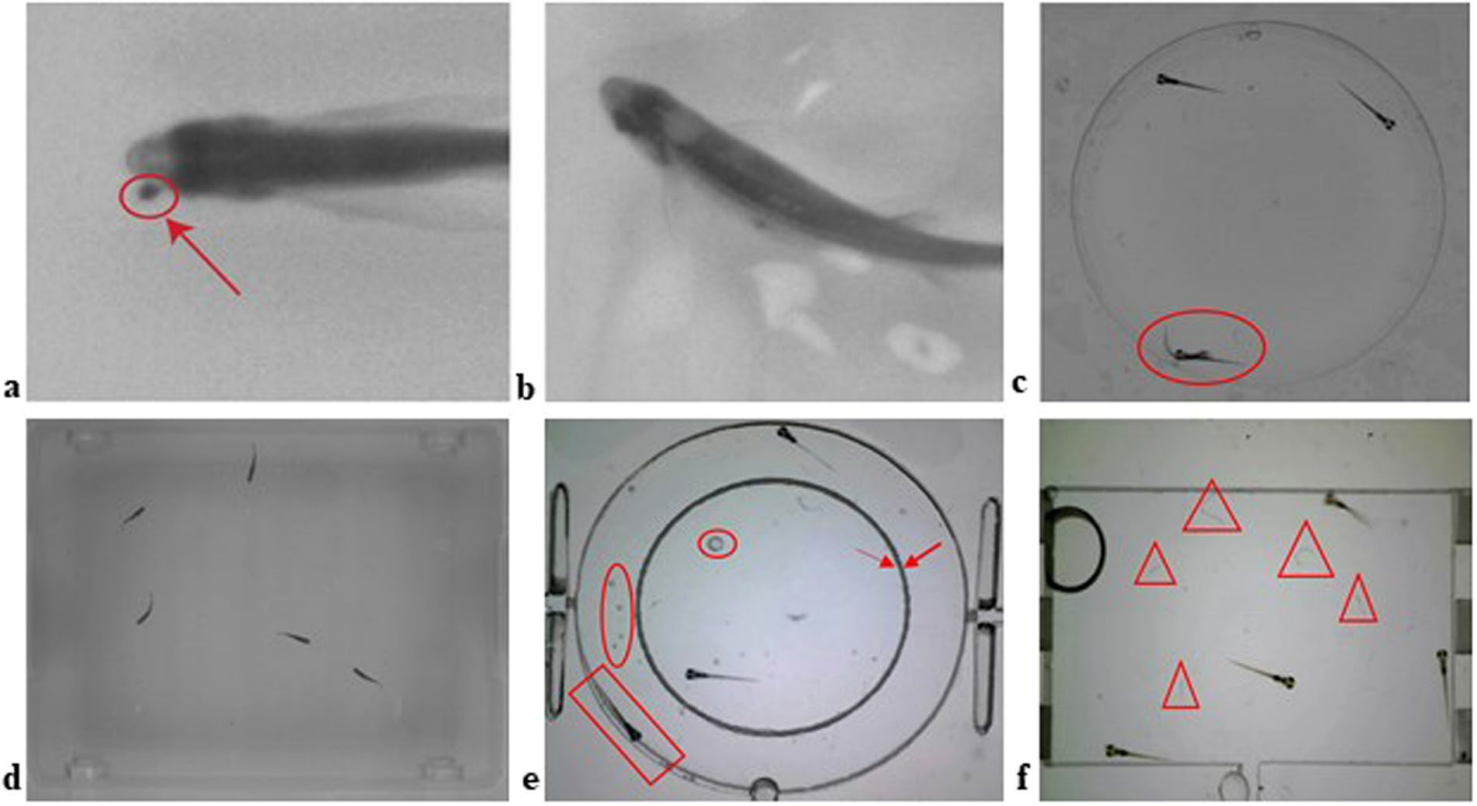
Video frame examples in variant imaging conditions: (**a**,**b**) Small water impurities as indicated by the red circle in (**a**) and water reflection or ripple in (**b**) can affect the head detection claimed by^7^; (**c**) Frame example with larvae occlusion, which will not been seen when the larvae are separated in petri dish plates; (**d**) idTracker^5^ required frame input with clear tank edges, and large size ratio between adult fish and the container; (**e**) Frame example with labelling as indicated by the red arrows, water bubbles as highlighted by the red circles, and larvae with low intensity contrast between the well edge shadow as shown by the red rectangle; (**f**) Frame example with small water particles as shown by the red triangles.

Though the widely used LSRtrack and LSRanalyse^1^, VideoHacking^4^ and the state-of-the-art approach in^8^ explored zebrafish larvae tracking, the video input must be under strict constraint imaging conditions. As reported in^1^ and^7^, even small impurities inside the water (as shown in Fig. 1a) and lighting reflections (as shown in Fig. 1b) will affect the tracking result. In addition, the small size difference between the zebrafish larvae and the petri dish (as shown in Fig. 1c) and that of adult zebrafish with the fish tank (as shown in Fig. 1d) causes water impurities such as water bubbles (as shown by the red circles in Fig. 1e), excretion, small particles (as shown by the red triangles in Fig. 1f) that are not usually detectable in adult fish experiments but inevitably affect the detection of zebrafish larvae. Strict input imaging conditions are impossible to maintain in practice: for example, even if a clean environment is originally used to house the organism, excretions produced by the organisms during the experiment can render it impossible to maintain a completely clean and transparent container background during long-term organism observation. IdTracker^5^ even explicitly defined the smallest acceptable size ratio between the zebrafish and the tank for creating the clear background environment required for the video data. In addition, these larvae tracking systems^1,4,8^ use a petri dish plate to separate individual zebrafish larvae, allowing only one zebrafish larvae in each petri dish to avoid overlapped and swapped trajectories that can result from multiple zebrafish larvae housed in one container (as shown in Fig. 1c). However, limiting experiments to one zebrafish per dish strictly constrains the research application as interaction and grouping behaviour cannot be studied.

The constraints on the input imaging conditions as required by existing systems are largely due to poor object detection and segmentation results from the input videos. Thus, improving the segmentation method will remove the need for input imaging constraints, where it has been shown that improving the segmentation accuracy can result in more reliable tracking performance^11^. However, this assumption of improving segmentation accuracy to enhance tracking performance has not yet been examined.

This paper investigates the novel adaptation of advanced computer vision techniques and multiple object tracking algorithms to develop an automatic, accurate and effective multiple zebrafish larvae tracking system using microscopic larvae videos, without any constraints on the input video imaging conditions. The proposed system is designed to segment and track the ‘bursty’ movement characteristics specific to zebrafish larvae, where the resultant tracking trajectories generated by the proposed system can then be used for further study, including the analysis of larvae movement characteristics. The performance of the proposed system is evaluated based on segmentation and tracking accuracy using a zebrafish larvae dataset also presented in this paper, and compared with the current state-of-the-art idTracker system^5^ and the off-the-shelf commercial Lolitrack system^6^, which allows for both single and multiple zebrafish larvae tracking. For reproducible research, the dataset generated for evaluation and the software for the proposed zebrafish larvae tracking and evaluation methods are publicly available online.

## Methods and Materials

**Figure 2:**
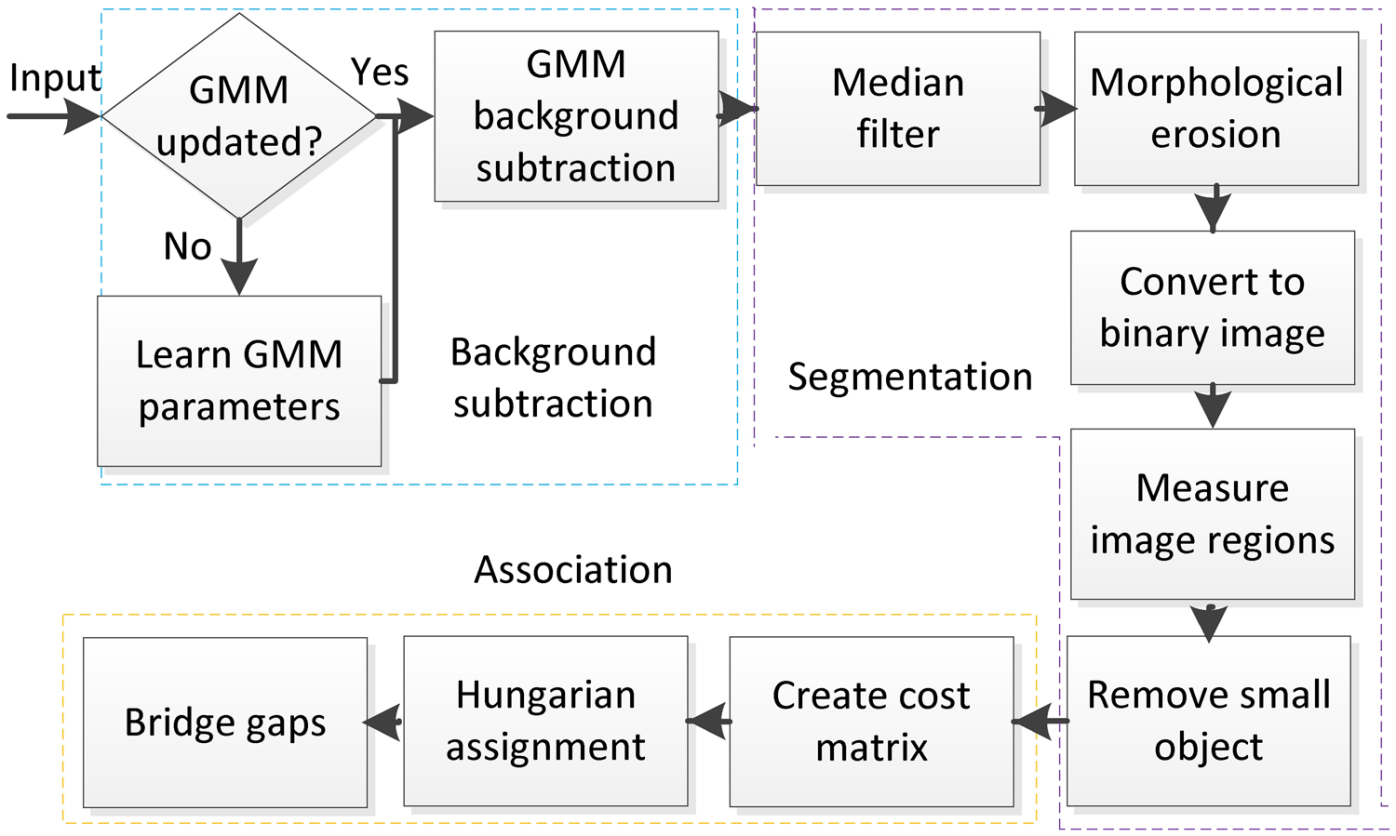
Overview of the proposed zebrafish larvae tracking system.

Figure 2 outlines the proposed automatic zebrafish larvae tracking system, which consists of multiple stages: background subtraction, zebrafish larvae segmentation, association or matching of the larvae between successive frames, followed by bridging any remaining gaps amongst the trajectory fragments.

### Background Subtraction

The efficient estimation and removal of the video frame background is essential for the zebrafish larvae tracking, in particular to address water impurities and ‘bursty’ movement characteristics. The water particles, ripples and bubbles (as shown in Fig. 1) when stirred up by the larvae tail beating produce motion data, which means that larvae detection methods based on motion and frame-to-frame differences cannot be applied^12^. As the movement of water impurities is relatively temporary compared to the larvae movement, the proposed system applies an improved adaptive Gaussian Mixture Model (GMM)^13^.

The distribution probability density model parameters for the GMM are calculated according to the mixing weight and Minimum Message Length (MML) criterion^13^, and the improved adaptive GMM introduces an exponential decay envelope shaped by the constant factor α to adapt to background changes. The decay envelope factor α strongly weights the pixel samples representing the temporary movement of these water impurities and illumination changes, to minimise the influence of this temporary movement and enable fast adaptation to background changes. Therefore, the zebrafish larvae regions in the video frames are distinguished as moving objects and segmented by subtracting the calculated background model from the original video frames. That is, in the proposed system there are no requirements on the video images to have a clear background, use transparent containers without edge shadows, or have high intensity contrast between the zebrafish larvae and the background.

The time interval, T, is the length of samples and is determined as per the work in^13^, where the first 500 frames of videos are used to estimate the GMM model parameters so as to obtain a consistent background model. In practice, however, there are many short microscopic zebrafish larvae videos where the number of frames is less than the required time interval, T. As a solution, duplicate video frames are added at the beginning of short videos to allow GMM model background estimation. This process is explained and illustrated in Supplementary Note in the dataset^14^.

To address the ‘bursty’ movement specific to zebrafish larvae, with sudden swimming locomotion interspersed with substantially stationary periods of little movement^10^, the applied GMM model^13^ in the proposed system adds flexibility to the description of the background by adaptively and recursively selecting the number of Gaussian components used to represent each pixel compared with traditional GMM models with one or a fixed number of components to model each pixel. To determine the number of Gaussian components, the Dirichlet prior^15^ and the MML criteria^13^ are used to select the number of components on the basis of the final value of the component mixing weights. The same approach and initialization settings used in^16^ are applied to recursively update the mixing weights for each new sample. For the initialisation stage, the approach and number of randomly generated components are taken from^16^, and the Dirichlet prior is applied. After each update, components holding a negative weight will be discarded by the MML criteria, with the components remaining taken as the number used in the model. The background model enables the removal of stationary background regions such as the zebrafish container and labels drawn on the petri dish; hence, unlike existing techniques, the proposed system is able to process larvae videos under practical experimental conditions.

However, if a larva in the video becomes static for some time, its body pixels will start to generate an additional stable cluster of pixels. But with the previously calculated background being occluded, the starting weight of the new stable cluster is very small. The cluster will only be classified to background model when its weight is larger than a threshold (referred to as *c*_*f*_ in^13^) when the larvae remains static for long enough, which will consistently increase the weight of the newly generated cluster. Thus, the detection period of larvae with no movement is extended for approximately $\mathrm{log}(1-{c}_{f})/\mathrm{log}(1-\alpha )$ frames as calculated in^13^.

### Organism segmentation

After background subtraction, the resultant image generally still contains distortion due to a scattering of small noise fragments that are detected by the GMM model as moving objects. Thus, to remove these noise fragments the proposed system applies a median filter with a 3 × 3 square moving window (the smallest available window size for removing small fragments) and morphological gray scale erosion^17^.

Water ripples evoked by zebrafish larvae movement are also often detected by the GMM model. Further, the ripples cannot be completely removed by the median filter and mathematical morphological operation because their relative region size is typically larger than the noise. For these distortions, a binary image/bitmap is firstly obtained from the grayscale image based on the global normalized threshold calculated using Otsu’s method^18^. Then, the system calculates the number of pixels from each connected component in the binary image, and estimates the average size across the regions in the image. Regions which are less than 20% of the average larvae size (based on the typical relative size of ripples evoked by the larvae) are then removed from the segmentation image. This 20% threshold is taken from the experiments in^5^, and empirical tests were conducted to verify this threshold as appropriate to the proposed system.

### Organism association between frames

After organism segmentation, the moving zebrafish larvae are associated or matched between successive frames to obtain organism tracking trajectories. A *n* × *m* matrix **D** is created to annotate the cost of associating source objects $O=\{{O}_{1},{O}_{2},\ldots ,{O}_{n}\}$ in the frame *t* to the target objects $T=\{{T}_{1},{T}_{2},\ldots ,{T}_{m}\}$ in the frame *t* + 1:

1 $${\bf{D}}(O,T)=(\begin{array}{cccc}{d}_{{O}_{1},{T}_{1}} & {d}_{{O}_{1},{T}_{2}} & \ldots  & {d}_{{O}_{1},{T}_{m}}\\ {d}_{{O}_{2},{T}_{1}} & {d}_{{O}_{2},{T}_{2}} & \ldots  & {d}_{{O}_{2},{T}_{m}}\\ \vdots  & \vdots  & \ddots  & \vdots \\ {d}_{{O}_{n},{T}_{1}} & {d}_{{O}_{n},{T}_{2}} & \ldots  & {d}_{{O}_{n},{T}_{m}}\end{array})$$

where *n* is the detected number of zebrafish larvae in the frame *t*, and *m* is the number of zebrafish larvae segmented in the successive frame *t* + 1. The element ${d}_{{O}_{i},{T}_{j}}$ in the matrix denotes the cost to connect the *i*-th object in the frame *t*, to the *j*-th object in the frame *t* + 1. The value of ${d}_{{O}_{i},{T}_{j}}$ is calculated as the Euclidian distance from the source object to the target object based on the centroids of the segmented regions in Cartesian coordinates *O*_*i*_(*x*_*i*_, *y*_*i*_) and *T*_*j*_(*x*_*j*_, *y*_*j*_), as given by:

2 $${d}_{{O}_{i},{T}_{j}}={({x}_{j}-{x}_{i})}^{2}+{({y}_{j}-{y}_{i})}^{2}$$

The frame-to-frame organism assignment based on the cost matrix is performed using the Muncres implementation of the Hungarian algorithm^19^, which searches for unique assignments i.e., assigns source object *i* to only one target object *j* in the secondary frame. The assignment is based on the global minimum of the smallest sum of squares distance amongst all of the possible associations, and allows $n\ne m$ in case of organism detection failure or larvae occlusion. Water impurities and well edge shadows may still remain in the binary bitmap, and to avoid these remaining noise fragments the maximum value $\max (dis{t}_{GT})$ of organism displacement extracted from the tracking ground truth is defined as the distance threshold^19^. In the cases where ${d}_{{O}_{i},{T}_{j}}$ is greater than the distance threshold, the value of ${d}_{{O}_{i},{T}_{j}}$ in the cost matrix **D** is set to *Inf* before mapping association.

In the cases where zebrafish larvae fail to be detected or segmented in one frame but reappear in subsequent video frames, a ‘gap’ in the moving trajectory of this object will appear at the frame where the zebrafish larvae detection initially failed, with a resulting new trajectory created from the frame where the zebrafish reappears. Scenarios of multiple object occlusion^7^ and misdetection of long-term stationary larvae objects can generate such trajectory ‘gaps’. Thus, in the proposed tracking system a ‘gap bridging’ stage is performed using the nearest neighbour algorithm^20^ to connect trajectory fragments and improve the inter-frame organism association. However, the trajectory gap will not be connected if the squared distance calculated between the two frames of trajectory fragments is greater than the distance condition calculated as $1.2\ast {\max }^{2}(dis{t}_{GT})$. The ratio of 1.2 is extended by 20% beyond unity to set a margin for rebound, similar to the threshold *ξ* in the gap filling stage of^7^, with the trajectory at that frame recorded as an error.

### Zebrafish larvae video dataset

Largely due to the time and manual labour required to generate ground truth segmentation and tracking, standard datasets for benchmarking moving objects in video sequences are still emerging. For this work, the authors have not yet discovered any publicly available zebrafish larvae video segmentation and tracking datasets. Thus, this paper generated a dataset with segmentation and tracking ground truth annotated per frame, labelling the zebrafish and background for segmentation and tracking accuracy evaluation.

Wild zebrafish embryos (Danio rerio) were incubated at 28 °C in a Petri dish filled with an E3 medium. Any debris and unfertilised embryos were manually removed three hours post-fertilization (hpf). Five days post-fertilization, the larvae were obtained from hatched zebrafish embryos. For data acquisition, zebrafish larvae were transferred to poly (methyl methacrylate) (PMMA) housing wells. Low frame rate videos were recorded with a Dino-Lite AD7013MT microscope at frame rates of 14 or 15 fps. High frame-rate videos were captured by an Imaging Development Systems (IDS) UI-3360CP-C-HQ microscope, with a high resolution 12.5 mm focal lens.

The dataset consists of 10 video sequences with 3056 frames in total, with various durations and imaging conditions as summarized below (the detailed sequence information and the code to facilitate the ground truth generation are freely available online^14^):

- Video durations from 110–759 frames with frame rates of 14–15fps (seq. 1–6, 8–9) and a high frame rate of 117fps (seq. 7, 10)
- Single zebrafish larvae (seq. 5, 6) and multiple zebrafish larvae (seq. 1–4, 7–10) swimming in round (as shown in Fig. 1c,e) or square (as shown in Fig. 1d,f) well containers
- Clear and obstructed well containers e.g., well edge shadowing (seq. 1, 10), water particles (seq. 2, 4–8), water bubbles (seq. 8), labels (seq. 4, 8–9)

### Segmentation evaluation metrics

This paper uses three standard metrics and proposes a new metric to numerically quantify the segmentation performance by calculating dissimilarity with the manually generated ground truth. Let $S$ and $\bar{S}$ denote the segmentation ground truth and the result of a segmentation algorithm for image *X* = {*x*_1_, *x*_2_, …, *x*_*N*_} of *N* pixels. Then, denote a pixel *x*_*i*_ in the detected object region *C*(*S*, *x*_*i*_) and *C*($\bar{S}$, *x*_*i*_), in the ground truth and algorithm result, respectively. The three standard segmentation metrics are defined in Equations 3–5 as:

3 $$Recall=\frac{|C(S,{x}_{i})\cap C(\overline{S},{x}_{i})|}{|C(S,{x}_{i})|}$$

4 $$Precision=\frac{|C(S,{x}_{i})\cap C(\overline{S},{x}_{i})|}{|C(\overline{S},{x}_{i})|}$$

5 $${F}_{measure}=\frac{{\rm{2}}\ast (Recall\ast Precision)}{Recall+Precision}$$

6 $$SI={F}_{measure}-\frac{Nu{m}_{miss}}{2\ast Nu{m}_{GT}}$$

The proposed *Similarity Index* ($SI$) metric in Equation (6) accounts for the number of correctly segmented objects by penalizing missing objects or object occlusion. $Nu{m}_{miss}$ and $Nu{m}_{GT}$ are the number of objects missed, and objects detected in the ground truth, respectively. The recall and precision metrics estimate under-segmentation and over-segmentation, respectively. The ${F}_{measure}$ is a weighted calculation of the precision and recall.

### Tracking evaluation metrics

To enable the objective evaluation of tracking performance on the database, this paper employs the widely utilized standard Multiple Object Tracking (MOT) metric: Classification of Events, Activities and Relationships (CLEAR MOT)^21^.

CLEAR MOT consists of two metrics: Multiple Object Tracking Precision (MOTP), which estimates the location precision of all detected objects compared to that of the manually labelled zebrafish larvae positions in each frame (known as ground truth); and, Multiple Object Tracking Accuracy (MOTA), which measures the accuracy in tracking object trajectories (producing exactly one trajectory per object), and the ability to consistently label objects over time. Mathematically, the MOTP and MOTA metrics are represented as:

7 $$MOTP=\frac{{\sum }_{i,t}|{D}_{i,t}-G{T}_{i,t}|}{{\sum }_{t}{N}_{t}}$$

8 $$MOTA=1-\frac{{\sum }_{t}({m}_{t}+f{p}_{t}+mm{e}_{t})}{{\sum }_{t}{g}_{t}}$$

where |*D*_*i*,*t*_ − *GT*_*i*,*t*_| indicates the Euclidian distance between the pair-wise matched position of the *i*-th segmented object in the *t*-th frame (*D*_*i*,*t*_) and the position of this object in the ground truth (*GT*_*i*,*t*_), averaged by the total number of matches in the entire video sequence.

In the MOTA metric, *m*_*t*_, *fp*_*t*_, and *mme*_*t*_ for each frame *t* indicate the number of missed zebrafish detections, false positive segmentation (i.e., image noise fragment detected as zebrafish), and the swapping of identities for individual zebrafish larvae, respectively. *g*_*t*_ represents the total number of objects presented in frame *t*.

## Results

To evaluate the segmentation approach in the proposed tracking system, the proposed segmentation approach is compared with the segmentation method within idTracker^5^, and the well-known motion feature based optical flow^22^ and SIFT flow^23^ methods. The overall tracking accuracy of the proposed system is then compared with idTracker^5^, and the widely used commercial LoliTrack system^6^. All evaluation experiments were performed using the zebrafish larvae segmentation and tracking dataset presented in this paper, annotated with manually generated segmentation and tracking ground truth.

### Segmentation evaluation

**Figure 3:**
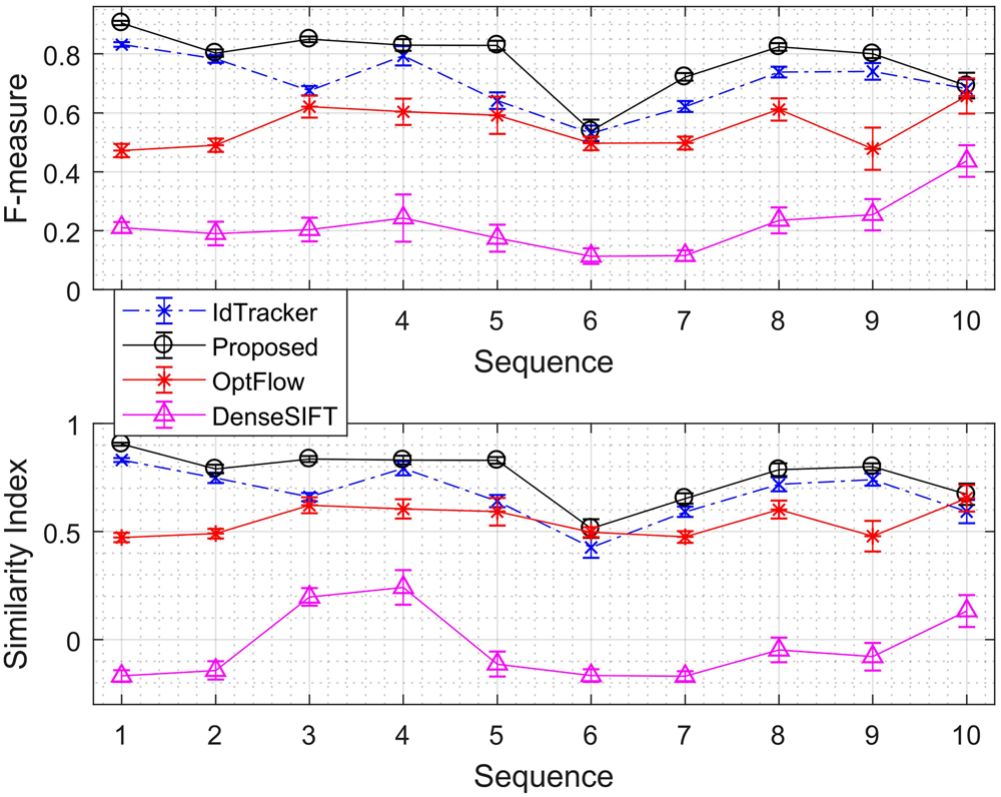
Segmentation accuracy over the 10 video sequences.

Figure 3 shows the average *F*_*measure*_ and *SI* scores presented with the 95% confidence intervals for each of the 10 video sequences in the dataset, presented in the order that the first sequence has clearest background and the 10th (last) sequence has the most complex background.

The results as seen in Fig. 3 show that the overall segmentation accuracy of the proposed method has an average 7.54%, 22.72% and 56.12% higher ${F}_{measure}$ than idTracker, optical flow and SIFT flow, respectively. The proposed approach also exhibits an 8.74% and 21.30% higher similarity index compared to idTracker, and optical flow, respectively, indicating an improved performance in relation to missing or occluded objects. In particular, the proposed method is more robust against challenging background environments, such as unclear zebrafish well containers with labels (as illustrated by seqs. 8 and 9). The robust segmentation accuracy as seen in Fig. 3 across the 10 videos under variant background conditions evaluated with the proposed system further shows that the segmentation performance does not depend on video input tested.

The sensitivity of the segmentation accuracy due to the tuning factor α was examined in^12^, where the range of α values evaluated showed a consistent and reliable segmentation performance. In turn, the robust segmentation accuracy seen in Fig. 3, which illustrates the 10 videos under variant background conditions evaluated with the proposed system shows that the segmentation performance does not depend on the video input tested. Further, optical flow, SIFT flow and idTracker respectively exhibit 1.95%, 2.45% and 0.47% more variance in all of the evaluation metrics studied, which suggests that the segmentation results are less reliable across the complex video sequences evaluated.

### Tracking accuracy evaluation

**Figure 4:**
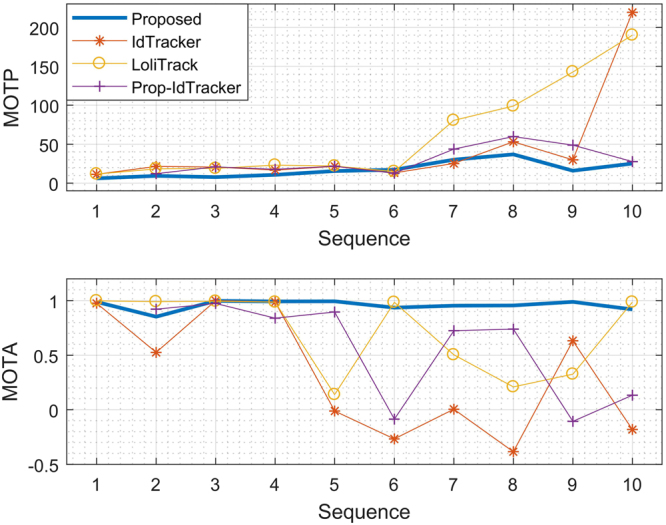
Tracking accuracy over the 10 video sequences.

Figure 4 summarises the tracking accuracy using the MOTP and MOTA metrics^21^ evaluated over the 10 video sequences, where the raw tracking accuracy for each video is provided in Supplementary Table S1. Seq. 1 has the clearest background, seq. 2–6 each have one type obstruction (well edge shadows, particles, particles, labels on well, and well edge shadows, respectively), and seq. 7–8 each have two types of obstruction, and seq. 10 has the most complex container background conditions. Both the proposed tracking system and existing tracking approaches perform reliably when the videos have restricted background conditions, as shown by the MOTP and MOTA values for seqs. 1–4 in Fig. 4. However, when the video conditions increasingly degrade from seqs. 5–10, the proposed system is more reliable than the existing systems as illustrated by the consistent performance of the proposed system compared with the existing systems as measured by both metrics from seqs. 5–10 in Fig. 4. Further, the proposed system exhibits the smallest position detection error, with a decreased overall error of 25.61 and 44.49 pixels using MOTP compared to idTracker and LoliTrack, respectively, and an increased accuracy of 31.57% and 27.2% using MOTA compared to idTracker and LoliTrack, respectively.

The proposed segmentation approach is applied as pre-processing to the idTracker system to determine the effect of the proposed segmentation approach on the overall tracking accuracy. The result of both the MOTP and MOTA values improved by 32.00% and 22.91%, respectively, compared with the original idTracker system. The proposed background subtraction and segmentation processing also removes the need to constrain the input zebrafish larvae video imaging conditions, and enables the testing of videos under realistic experimental conditions using idTracker. That is, researchers who already use idTracker can apply the proposed segmentation method as pre-processing to obtain tracking results of higher accuracy using the existing idTracker system, with video data in unconstrained imaging conditions.

**Figure 5:**
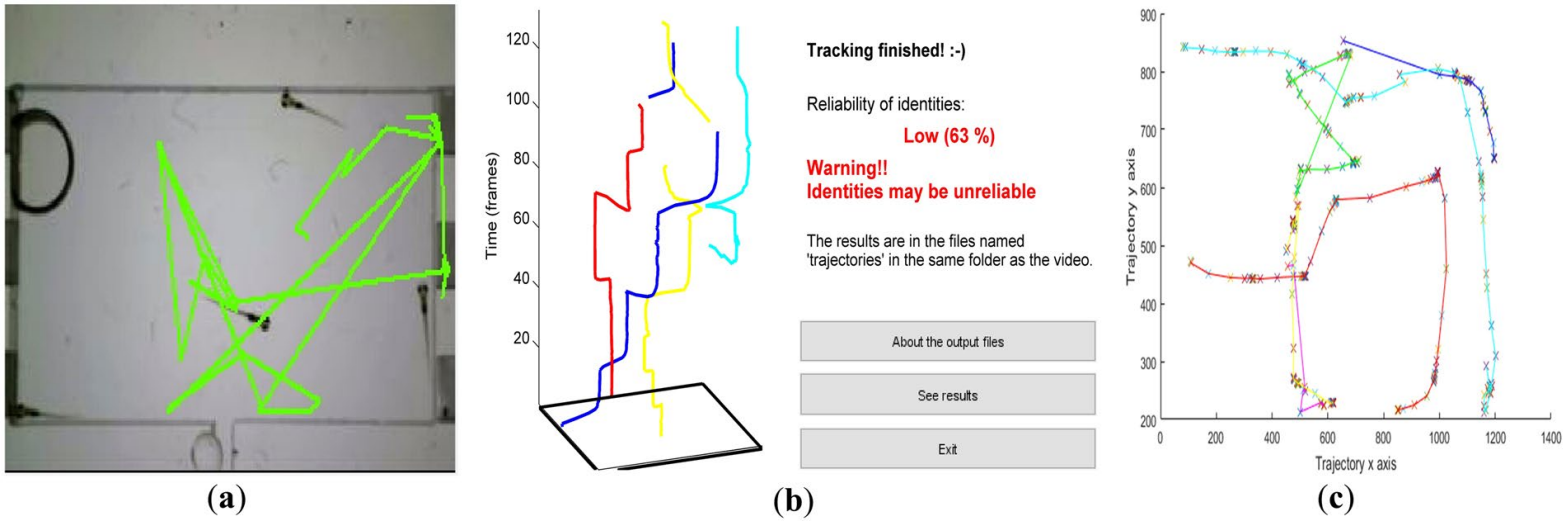
Visual example comparing tracking trajectories. (**a**) Lolitrack; (**b**) idTracker; (**c**) Proposed system.

Figure 5 is a visual example of the tracking trajectory obtained for seq. 4 by LoliTrack, idTracker and the proposed system. It can be seen that the proposed tracking system exhibits the most complete tracking trajectories estimated for realistic experimental conditions. In contrast, LoliTrack (Fig. 5a) detects the well edge shadow as zebrafish due to their similar intensity values, whilst idTracker system (Fig. 5b) produces many trajectory gaps primarily caused by the false detection (as shown by the light blue line) of larvae objects due to their ‘bursty’ locomotive characteristics and small size differentiation with impurities inside water. The resulting identity estimated from idTracker is therefore also not reliable, with an estimated reliability of identity of 63% calculated according to the trajectory analysis of idTracker as shown by Fig. 5b.

Supplementary Table S2 summarises the total number of individual identities swapped across each tested video. The proposed system exhibits the smallest identity swapping rate, with 24.32% less identity swap than the idTracker system. In addition to the proposed segmentation method exhibiting a consistently higher accuracy segmentation than idTracker as shown in Fig. 3, applying the proposed segmentation method to idTracker reduces the zebrafish larvae misdetection and false positive rates, as shown in Fig. 4. However, the identity swapping rate is doubled as shown by Supplementary Table S2, due to the generated binary foreground images providing limited intensity information for idTracker to generate the required fingerprint.

## Conclusion

Compared to the tracking of adult zebrafish in microscopic videos, the dynamic ‘bursty’ locomotive characteristics and complex video imaging conditions of zebrafish larvae due to their small size relative to background imaging artifacts poses many different challenges for the tracking of multiple zebrafish larvae. This paper proposes a zebrafish larvae tracking system for both single and multiple zebrafish larvae under complex video conditions, applying an adaptive GMM probability density model, median filter and morphological operations to segment larvae objects from the background, and Hungarian assignment for tracking. Comparisons with existing state-of-the-art biological small organism tracking systems illustrated the accuracy and efficiency of the proposed system, where the proposed system also removes the strict limitations on input video imaging conditions to enable the testing of unconstrained experimental videos. Further, the proposed background subtraction and segmentation approaches applied alone as pre-processing to existing tracking systems (such as idTracker) improve the multiple organism tracking accuracy by up to 32%. This is due to decreased zebrafish larvae misdetection and false positive rates; however, the identity swapping rate may increase if the identity is generated using intensity variance information, such as the approach used in idTracker. The immediate future work is in evaluating the proposed tracking system for other biological organisms, including adult zebrafish. Together with the increased size and intensity contrast, the continuous swimming movements of adult zebrafish provide consistent motion features that can be easily captured by the adaptive GMM model and subsequent object tracking.

### Data availability

The datasets generated during and/or analysed during the current study are available in the GitHub repository, https://github.com/Xiao-ying/-moving-zebrafish-larvae-segmentation-dataset-/tree/master/Data.

## Electronic supplementary material

Supplementary Information
