# Supplementary material for: Automatic multiple zebrafish larvae tracking in unconstrained microscopic video conditions
Source: Sci Rep. 2017 Dec 14;7:17596. doi: 10.1038/s41598-017-17894-x (PMC5730596; doi:10.1038/s41598-017-17894-x)
Supplement: Supplementary file 1 — Supplementary Information [file 41598_2017_17894_MOESM1_ESM.pdf]

Automatic multiple zebrafish larvae tracking in unconstrained microscopic video conditions

Xiaoying Wang, Eva Cheng, Ian S. Burnett, Yushi Huang, Donald Wlodkowic

Supplementary Table S1

Table S1. Multiple object tracking accuracy for the systems evaluated

No.	MOTP (pixels)				MOTA			
	idTracker	Segment + idTracker	Loli Tracker	Propose d system	idTracker	Segmen+ idTracker	Loli Tracker	Propose d system
1	11.388	NaN*	11.662	6.346	1.000	NaN*	0.990	0.988
2	21.434	12.176	18.395	15.024	0.541	0.922	0.921	0.853
3	20.648	20.879	18.854	8.113	1	0.975	0.985	0.998
4	16.728	17.665	23.208	10.669	0.987	0.840	0.987	0.993
5	21.545	21.727	21.890	15.525	-0.01	0.895	0.139	0.994
6	13.151	12.562	15.020	12.786	-0.27	-0.0864	0.914	0.936
7	25.230	43.746	80.630	30.082	0.005	0.725	0.504	0.954
8	53.096	59.666	98.936	36.901	-0.39	0.739	0.209	0.956
9	29.921	48.739	142.834	15.960	0.673	-0.1071	0.327	0.989
10	219.329	27.532	189.975	25.121	-0.18	0.133	0.906	0.920
Average	43.247	29.410	62.14	**17.65**	0.33	0.56	0.69	**0.96**

* No valid data generated due to the running error when testing sequence 1.

Supplementary Table S2

Table S2. Total number of swapped individual identities

Mismatch ratio				
No.	idTracker	Segment + idTracker	Loli Tracker	Proposed
1	6	NaN	2	0
2	2	3	4	3
3	0	0	0	0
4	4	0	0	0
5	3	2	6	0
6	1	1	1	2
7	2	1	3	1
8	11	41	24	14
9	4	17	3	0
10	4	8	0	8
**Average**	3.7	8.1	4.3	**2.8**

Supplementary Note
Building training samples for a short video

October 31, 2017

For a short video where the length is less than the training sample required, duplicate video frames are added to the beginning of the video sequence to estimate background model. Fig. 1 illustrates the process of constructing the training samples from a short video.

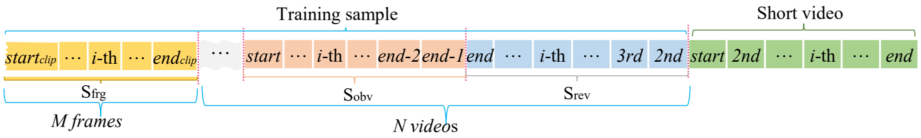

Figure 1: Building training samples for a short video.

The green frame series in Fig. 1 indicates the original time-lapse zebrafish larvae microscopic short video $\mathbb{V}$. The frames in front of this video are its training sample $\mathbb{S}$, used to estimate the background model parameters of $\mathbb{V}$. The set of training samples $\mathbb{S}$ consists of: S_{obv}, frames from the original short video $\mathbb{V}$ in obverse order from $[start, end-1]$ frames, shown by the pink frame series; S_{rev}, frames from the original video short video $\mathbb{V}$ in reverse order from $[end, 2nd]$ frames, shown as the light blue; and, S_{frg}, the video fragment shown in orange. The obverse order frame series S_{obv} and reverse order frame series S_{rev} are alternately linked to each other to construct the training sample, and the last frame to connect to the original video is the reverse order frame to ensure a smooth background transition between the last frame in the reverse order frame series to the first frame of the original microscopic short video.

To construct a training set with length of L_{sample} images, a video fragment, S_{frg}, taken from the short video studied will be added to the training set for the set length requirement when the S_{obv} and S_{rev} series do not have exact L_{sample} images.

The required number of videos, N and number of frames, M, are calculated using Eq. (1) and Eq. (2).

$$N = L_{sample} \backslash N_{frames} \tag{1}$$

where the MATLAB built-in function idivide is applied for the integer division with fractional quotients being rounded toward negative infinity to the nearest integer for implementation.

$$M = L_{sample} - L_{sample} \backslash N_{frames} \tag{2}$$

Based on the required number of videos N (in obverse order and reverse order), the video fragment S_{frg} is constructed according to Eq. (3).

$$S_{frg} = \begin{cases} \{S_{rev}(i) \mid i = M+1 \ , \ \ldots \ , \ 3, \ 2\}, & \frac{N}{2} \ is \ even \\ \{S_{obv}(i) \mid i = 2 \ , \ \ldots \ , \ 3, \ M+1\}, & \frac{N}{2} \ is \ odd \end{cases} \tag{3}$$
